# Supplementary material for: Incipient charge order observed by NMR in the normal state of YBa2Cu3Oy
Source: Nat Commun. 2015 Mar 9;6:6438. doi: 10.1038/ncomms7438 (PMC4366503; doi:10.1038/ncomms7438)
Supplement: Supplementary Information — Supplementary Figures 1-8 and Supplementary References [file ncomms7438-s1.pdf]

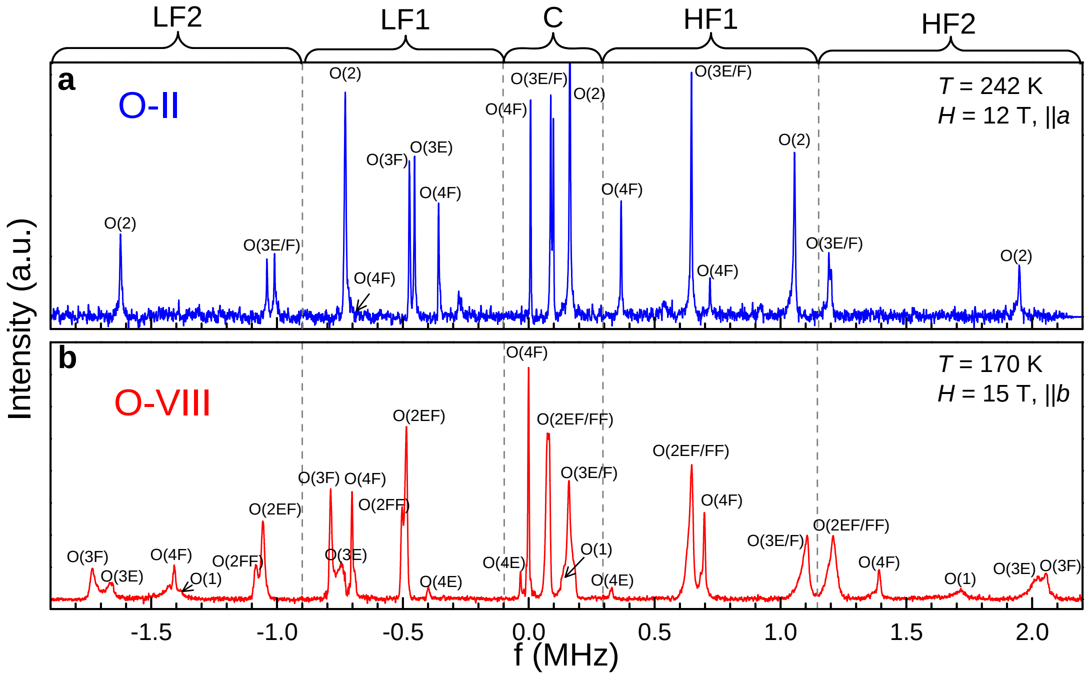

Supplementary Figure 1 | ^{17}O NMR spectra in YBCO ortho-II and ortho-VIII.

a, **b**, spectra for the two samples reported in this study. LF1 (LF2) stand for the first (second) low-frequency satellites of planar O(2) and O(3) sites. C stands for central lines. HF1 (HF2) stand for the first (second) high-frequency satellites of planar O(2) and O(3) sites. For the ortho-II sample, the O(4E) lines are not seen in this particular spectrum, due to the use of a repetition time much shorter than their T_1 value. Also, the chain O(1) signal is not seen in ortho-II presumably because the longer chain length produces broader lines and/or shorter T_2. All the parameters (magnetic hyperfine shift K, quadrupole coupling ν_Q and asymmetry parameter η) of all sites are quantitatively consistent with previous works (1,2).

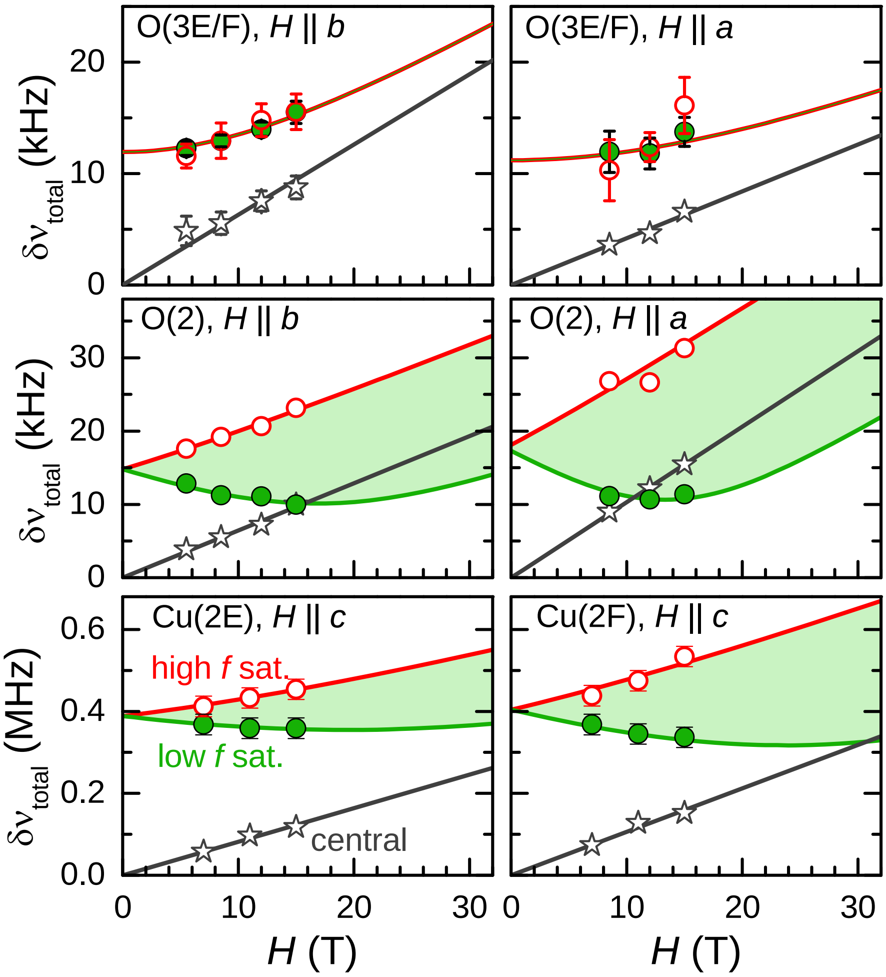

Supplementary Figure 2 | Field dependence of the linewidth in YBCO ortho-II.

The different dependence for the high-frequency (red) and low-frequency (green) satellites is perfectly accounted for by a simple model (continuous lines) of linearly-coupled electric-quadrupole and hyperfine-magnetic broadening mechanisms. Note that for the O(3) sites of this sample, the quadrupole broadening occurs without any magnetic broadening coupled to it.

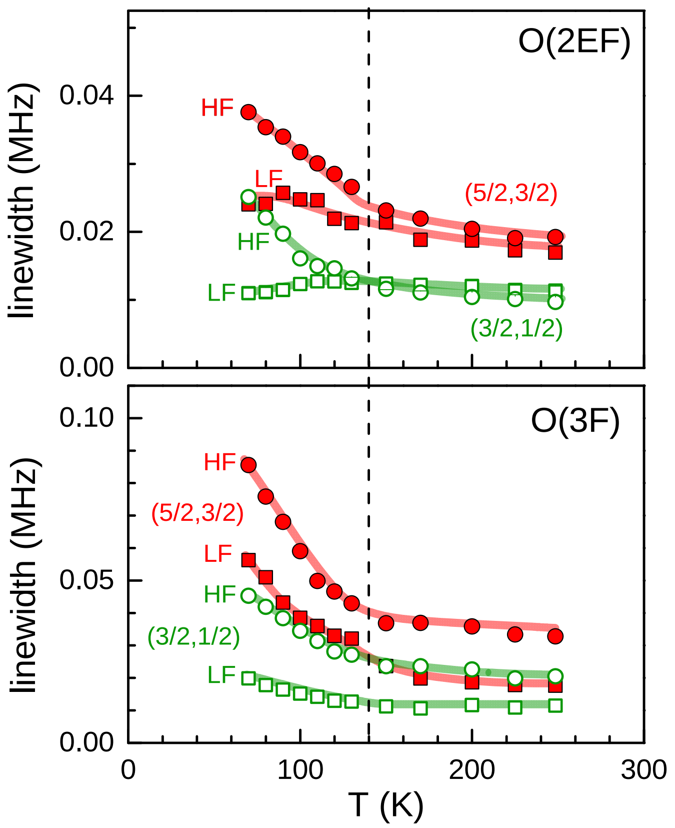

Supplementary Figure 3 | Raw ^{17}O linewidth data for YBCO orho-VIII.

HF and LF stand for low-frequency (3/2, 1/2) and high-frequency (5/2, 3/2) satellites. Although data above 150 K are not temperature independent, there is a clear temperature crossover defining $T_{onset} \approx 140$ K (dashed line). Thick traces through symbols are guides to the eye. The difference between O(2EF) and O(3F) data constitutes direct evidence of inequivalence of the two sites.

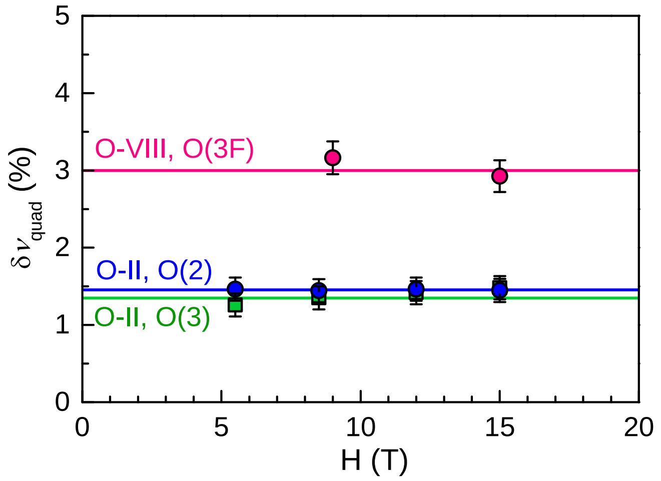

Supplementary Figure 4 | Absence of field-dependence of the quadrupole broadening.

Data for ortho-II were obtained at T = 60 K with $H||b$. Data for ortho-VIII were obtained T = 80 K with $H||b$.

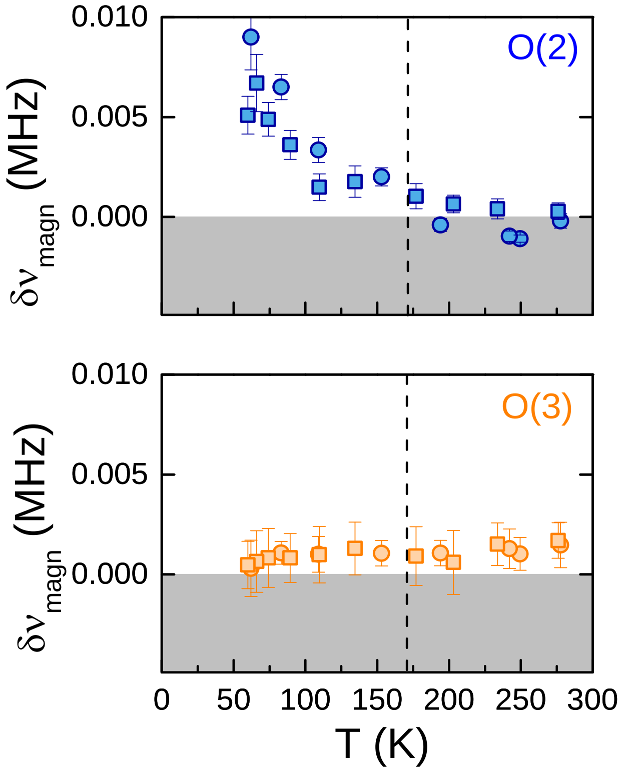

Supplementary Figure 5 | CDW-induced magnetic broadening in YBCO ortho-II.

Circles correspond to *H*||*a* and squares to *H*||*b*. The results are identical for the two field orientations.

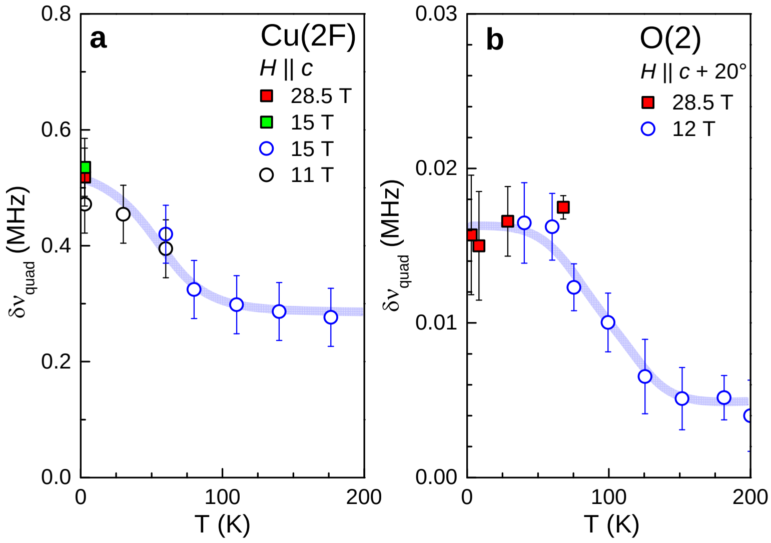

Supplementary Figure 6 | Persistence of the broadening below T_{charge} in YBCO ortho-II.

Filled squares correspond to the quadrupole-width of the split lines in the charge-ordered state (data for each of the split line are not shown for clarity and they are identical to within ~10%, so the average value is plotted here). Open circles correspond to data shown in Fig. 2. If the normal-state broadening (open circles) were an unresolved line splitting eventually becoming apparent at T_{charge}, the split lines just below T_{charge} (filled symbols) would be not narrower than the unsplit line at T_{charge}. This is not what is observed here: the broadening persists below T_{charge}. Note that the data point for O(2) at 28.5 T and 68 K may be slightly overestimated if a small splitting is actually present: the fit with two lines gives a null splitting.

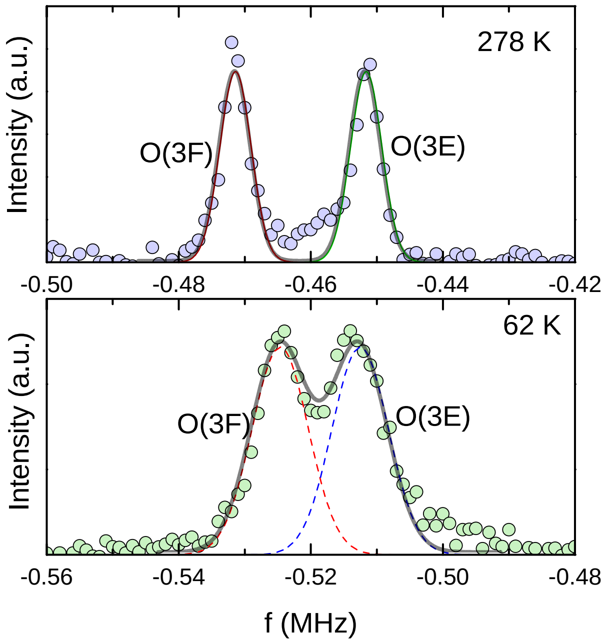

Supplementary Figure 7 | Identical width of O(3E) and O(3F) sites in YBCO ortho-II.

No difference is detected between these two sites throughout the normal state.

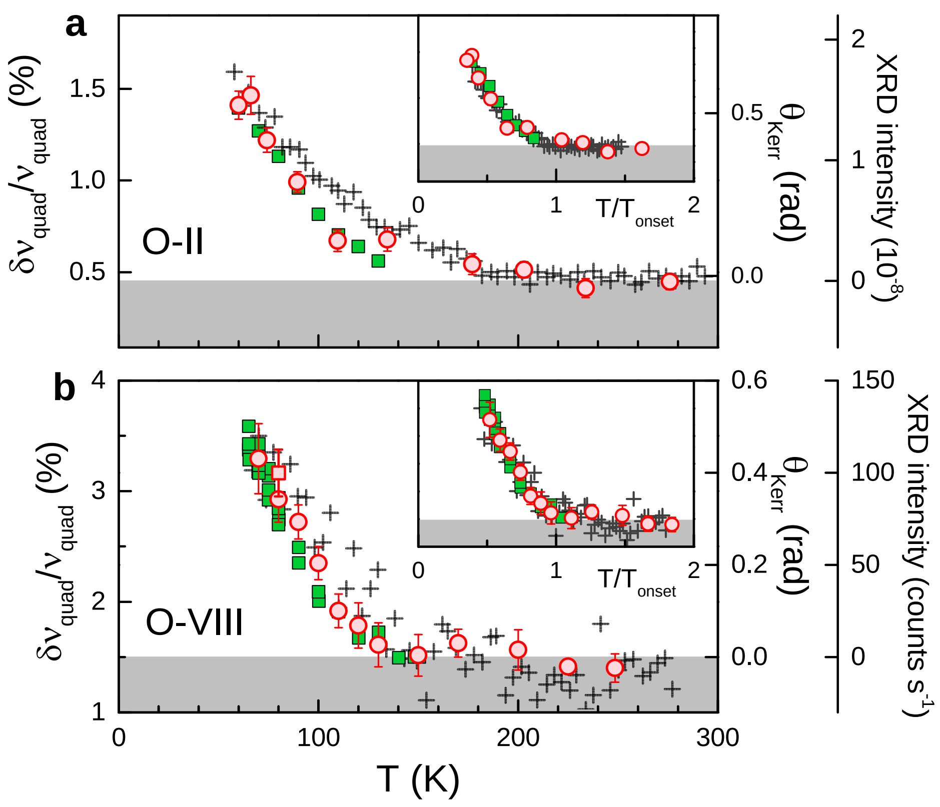

Supplementary Figure 8 | Connecting NMR, XRD and Kerr-effect data.

a,**b** quadrupolar part of the NMR broadening, X-ray scattering intensity and Kerr angle in ortho-II and ortho-VIII samples. Insets show the same data, in identical vertical axes, as a function T/T_{onset}. The scaling is made using Kerr-effect data (3) as a reference. Differences of T_{onset} values appear to arise mainly from small differences in the hole-content of the studied samples. **a**, NMR data (red symbols) for $YBa_2Cu_3O_{6.56}$ (T_{onset} = 170 K, T_c = 59.8 K), X-ray data (green squares, ref. 4) in $YBa_2Cu_3O_{6.55}$ (T_{onset} = 155 K, T_c = 61 K) and Kerr-effect data (crosses) in $YBa_2Cu_3O_{6.5}$ (T_{onset} = 200 K, T_c = 59 K) **b**, NMR data (red symbols) in $YBa_2Cu_3O_{6.68}$ (T_{onset} = 140 K, T_c = 67.8 K), X-ray data (green squares, ref. 5) in $YBa_2Cu_3O_{6.67}$ (T_{onset} = 135 K, T_c = 67 K) and Kerr-effect data (crosses) in $YBa_2Cu_3O_{6.67}$ (T_{onset} = 150 K, T_c = 65 K). The shaded area represents the high temperature background.

Supplementary references

1. Horvatić, M. *et al.*, Nuclear-spin-lattice relaxation rate of planar oxygen in $YBa_2Cu_3O_{6.52}$ and $YBa_{1.92}Sr_{0.08}Cu_3O_7$ single crystals. *Phys. Rev. B* **48**, 13848–13864 (1993).

2. Yamani, Z. *et al*. Cu NMR study of detwinned single crystals of Ortho-II YBCO6.5. *Physica C* **405**, 227–239 (2004).

3. Xia, J. *et al.* Polar Kerr-effect measurements of the high-temperature $YBa_2Cu_3O_{6+x}$ superconductor, evidence for broken symmetry near the pseudogap temperature. *Phys. Rev. Lett.* **100**, 127002 (2008).

4. Blanco-Canosa, S. *et al*. Momentum-dependent charge correlations in $YBa_2Cu_3O_{6+\delta}$ superconductors probed by resonant X-ray scattering: evidence for three competing phases. *Phys. Rev. Lett.* **110**, 187001 (2013).

5. Chang, J. *et al*. Direct observation of competition between superconductivity and charge density wave order in $YBa_2Cu_3O_{6.67}$. *Nature Phys.* **8,** 871–876 (2012).
